# Supplementary material for: Using large language models to accelerate communication for eye gaze typing users with ALS
Source: Nat Commun. 2024 Nov 1;15:9449. doi: 10.1038/s41467-024-53873-3 (PMC11530652; doi:10.1038/s41467-024-53873-3)
Supplement: Supplementary file 1 — Supplementary Information [file 41467_2024_53873_MOESM1_ESM.pdf]

Supplementary Information: Using Large Language Models to Accelerate Communication for Eye Gaze Typing Users with ALS

Ethics and Societal Impact

Techniques that improve AAC applications, such as the LLMs for assisting eye gaze typing studied in this paper, has the potential to significantly enhance quality of life, increase independence and social participation[1] of people living with communication and motor disabilities. Risks of abbreviation expansion and word replacement include insufficiently-personalized predictions being chosen by users during communication, which can potentially reduce the speaker's autonomy and authentic self-expression, which people e.g. with ALS[2] value highly. Another risk is that frequent incorrect or insufficiently-personalized predictions may increase effort required to edit minor errors in order to match the AAC users' personal vocabularies, dialects, and language styles, and inadvertently increase fatigue. This aspect awaits studies in a larger cohort of AAC eye-gaze typers with greater diversity in ethnic and educational backgrounds than the small number of AAC user-study participants in the current study.

1 Fine-tuning and offline evaluation of large language models

To synthesize examples for abbreviations with complete keywords, we determined the number of keywords in a sentence by using a uniform distribution [1, 3], while ensuring that at least one word of the sentence is preserved as a non-keyword (the initial). To synthesize examples for abbreviations with incomplete keywords, we randomly selected the number of keywords from a sentence by using the [1, 5] uniform distribution, also ensuring that not all words of a sentence are selected as keywords. For a given word selected as the incomplete keywords, we limited the number of letters in its abbreviation to N_L, with N_L following a uniform distribution [2, 5]. An equal number of examples were synthesized for the prefix and consonant incomplete-keyword schemes. Supplementary Table 1 summarizes the number of unique examples used in the fine-tuning of the KeywordAE LLMs. For KeywordAE v1, only the initials-only and complete-keyword examples were used; for KeywordAE v2, the dataset additionally included the incomplete-keyword examples, in order to support partially-spelled keywords.

To quantify the accuracy of the fine-tuned LaMDA on the AE task with the three types of abbreviation mentioned above, we evaluated the exact-match accuracy among top-5 predictions. For each AE input, 128 outputs were sampled from the temperature-based sampling if the total number of initials and keywords in the abbreviation was $\leq$5, else it was 256. The exact match was determined after text normalization including whitespace standardization, folding to lowercase, and omitting sentence-final punctuation. The sampling was based on a temperature of 1.0, which was determined on the Dev split to be the optimal sampling temperature value for AE. Panel A of Supplementary Figure 1 shows the evaluation results on the 2nd turn of the dialogues by using the first turn as the context. The general trends of relation between AE accuracy with abbreviation type and length is similar for other turns of the dialogues and when no dialogue context was used. On average, with initials-only abbreviation, 72.6% and 76.3% of the sentences could be expanded into the exactly-matching phrases (the single data point labeled blue in Supplementary Figure 1, Panel A) based on the v1 and v2 models, respectively. As expected and shown by the curves in Supplementary Figure 1A, incorporating keywords (both complete or incomplete) in the abbreviation progressively increased the percentage of successful expansion. The amount of improvement shows a monotonic relation with both the number of keywords and the number of characters used to represent each keyword. The v1 model showed slightly better accuracy over the v2 model for the initials-only and complete-keyword cases, which may be due to the v1 model's specialization on the more limited input format that doesn't include partially-spelled keywords.

To quantitatively illustrate the benefits of dialogue context, a similar evaluation was performed on all six turns of the TDC dialogues (test split), with and without dialogue context. The accuracies are plotted as a function of dialogue turn number in Panel B of Supplementary Figure 1. Comparing the sets of blue and orange curves, we see a clear and substantial boost to the v2 KeywordAE model's accuracy due to the dialogue context, which reflects the reduction in the space of possible phrases (given abbreviation) resulting from contextualizing on the previous turns of a dialogue. The panel also shows that this boost of dialogue context is seen for all three styles of abbreviations: initials-only, incomplete keywords, and complete keywords. The latter two show additional accuracy enhancement on top of the contextual boost, reflecting the expected trend that the fine-tuned LaMDA model predicts phrases more accurately when more characters become available, regardless of whether these additional characters form complete words. The effect of context and dialogue turns on the v1 KeywordAE model (the black and red curves in Supplementary Figure 1B), which doesn't not support incomplete keywords, closely resembles those on the v2 model.

Panel C of Supplementary Figure 1 shows the relation between AE accuracy and length of the phrase. We see a general decreasing trend for the accuracy of AE with increasing phrase length for initials-only AE, as well as incomplete and complete keywords. We can also see a strong trend in which augmenting the abbreviations with more characters, in the form of incomplete or complete keywords, boosts accuracy for all phrase lengths. The relations between AE accuracy and phrase length are similar between the v1 and v2 KeywordAE models.

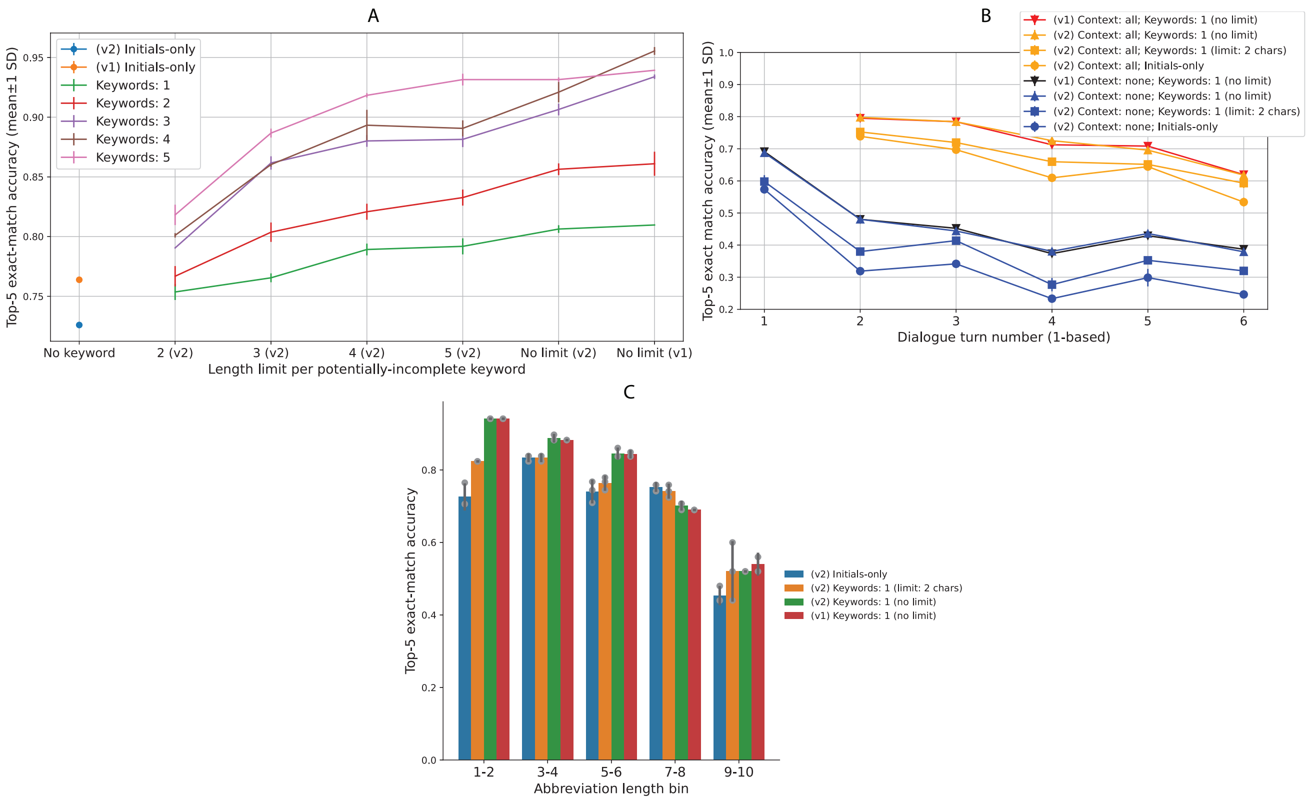

Supplementary Figure 1. Exact-match accuracy of the fine-tuned LaMDAs for abbreviation expansion, evaluated on the test split of the TDC corpus, under different levels of phrase and word-level abbreviation types and different amounts of dialogue context. The results are evaluated on the sentences from the test split of the Turk Dialogues Corrected (TDC) corpus for sentences of ten or fewer words and mid-sentence punctuation marks. The error bars who ±1 SD around the mean for 3 repeated evaluation runs. A: The five curves show the AE accuracy when different numbers of keywords (from 1 to 5) are included in the abbreviation, with the keywords chosen at random. The x-axis is the limit on the length of each potentially-incomplete keyword, so that the rightmost two ticks (labeled "No limit (v2)" and "No limit (v1)") correspond to using only complete keywords in the abbreviations. The single data point on the left plots the exact-match AE accuracy with initials-only abbreviations (i.e., no keyword). The complete-keyword accuracy of the dedicated v1 model slightly beat that of the v2 model. B: Representative relations between the accuracy of AE and dialogue turn number in a six-turn dialogue in the style of the TDC corpus, and how the relation is affected by the amount of dialogue context used when invoking the fine-tuned LaMDA for AE. For the orange curves, all previous dialogue turns were included as the context (e.g., when performing AE on dialogue turn #4, the content of the first three turns were used as the context). In each set of curves, three curves are shown, for initials-only AE, incomplete keyword AE (limiting keyword length to two characters, and complete keyword AE). The sets of blue and orange curves show the v2 model's accuracies without and with dialogue context, respectively. For comparison the accuracies of the v1 model for complete keywords are also shown (in black and red). C: AE accuracy as a function of phrase length as measured by the initials-only abbreviation length of a phrase (i.e., the number of words plus the number of mid-sentence punctuation). The first three different bar colors show the AE accuracies based on initials-only abbreviations, abbreviations with a single incomplete keyword limited to 2-character length, and a complete keyword, when used as input for the v2 model. The remaining bar color shows accuracies of the v1 model under complete keywords for comparison.

Dialogue Corpus	Abbreviation keyword type or FillMask	#(examples), tokens/example		
		Train	Dev	Test
Turk Dialogues Corrected (TDC)[3]	(i) Initials-only	8,590, 50.5±21.3	2,800, 50.6±21.4	2,800, 51.1±21.9
	(ii) Complete	7,948, 51.5±21.4	2,569, 51.7±21.6	2,545, 52.1±21.9
	(iii) Incomplete	15,158, 60.3±22.4	4,902, 60.3±22.9	4,898, 60.5±22.9
	(iv) FillMask	8,584, 46.2±19.7	2,800, 46.5±20.0	2,798, 46.8±20.3
Turk AAC[4]	(i) Initials-only	5,019, 21.5±5.3	559, 22.1±5.5	565, 21.0±5.0
	(ii) Complete	4,352, 22.9±5.1	490, 23.3±5.3	488, 22.3±4.8
	(iii) Incomplete	7,960, 29.9±8.1	904, 30.4±8.5	856, 29.0±7.9
	(iv) FillMask	5,019, 19.4±2.8	559, 19.7±2.9	565, 19.2±2.6
DailyDialog Corrected[5]	(i) Initials-only	123,650, 86.1±70.4	9,233, 84.4±66.4	8,318, 82.8±64.0
	(ii) Complete	99,202, 87.6±69.6	7,334, 85.9±64.9	6,612, 84.7±63.3
	(iii) Incomplete	196,286, 95.6±70.1	14,588, 93.8±66.0	13,198, 92.4±63.7
	(iv) FillMask	123,426, 82.2±69.7	9,211, 80.4±65.7	8,306, 79.0±63.2
Cornell Movie Dialogues[6]	(i) Initials-only	327,569, 63.1±64.0	41,636, 60.7±60.3	36,772, 64.4±68.2
	(ii) Complete	241,115, 67.4±65.1	30,268, 64.8±60.4	26,464, 68.8±68.0
	(iii) Incomplete	472,746, 74.8±65.7	59,124, 72.3±61.2	52,380, 75.7±67.4
	(iv) FillMask	323,834, 60.0±63.2	41,030, 57.8±59.5	36,057, 61.1±67.0

Supplementary Table 1. Composition of the data for fine-tuning and evaluating LaMDA for the AE and FillMask and tasks. For each dialogue corpus, the statistics for four subsets are shown: (i) no keyword, i.e., initials-only abbreviations; (ii) Complete keywords: initials with complete keywords, with the number of keywords per sentence distributed uniformed from 1 through 3; (iii) Incomplete keywords: initials with incomplete keywords, of both the prefix and consonant schemes, with the number of incomplete keywords per sentence distributed uniformly from 1 through 3 and the length limit of incomplete keyword uniformly distributed from 2 through 5; (iv) FillMask examples. (i) - (ii) were used to fine-tune LaMDA for KeywordAE v1 (i) - (iii) were used in KeywordAE v2 fine-tuning; and (iv) was in FillMask fine-tuning. The tokens/example column shows the mean lengths of the examples and their standard deviations based on the SentencePiece tokenizer[7] with 32,000 vocabulary items.

These results form the basis for designing the SpeakFaster UI (Fig. 1) that allows users to amend abbreviations that fail AE by adding more and more characters. The fact that even with five fully-spelled keywords, only 93.1% of the phrases could be expanded correctly is one of the motivations for allowing the user to use the additional remedying approach of FillMask and the always-available option of spelling out the words in full in the SpeakFaster UI.

Supplementary Figure 2 shows the results of evaluating the accuracy of the fine-tuned FillMask LaMDA model on sentences with randomly selected and masked words from the test split of the TDC dataset. Stop words (i.e., high-frequency words such as "the" and "for") have significantly higher prediction accuracy than non-stop words. As in the AE evaluation results (cf. Fig. 1, Panels A - B), incorporating conversational context increased the FillMask accuracy for both the stop and non-stop word categories. However, the benefit of context was much more pronounced for non-stop words (7 - 11 percentage points) than for stop words ($\leq$1 percentage points).

2 Detailed offline simulation results

The simulation results shown in the main article were based on utilizing all available conversational context. Specifically, when performing AE or FillMask on the n-th turn of a dialogue in the TDC corpus (test split) that we use for simulation, the 1st through (n-1)-th turns of the dialogues are fed to the fine-tuned LLMs to provide them with the maximal context for performing their respective inference tasks. However, in real-life situations, due to the constraints related to privacy, system capabilities, and the modality of text and speech communication, the contextual information may not always be available or accurate. The orange curve in Supplementary Figure 3 shows the KSR when no conversational context is used for inference with the fine-tuned LLMs, which show substantial decreases (10 percentage points or more) compared to the corresponding KSRs obtained under full context. This pattern is seen for all three simulation strategies (1, 2, and 2A), which were based on different degrees of utilization of the keyword AE and FillMask features. This observation corroborates the aforementioned evaluation results on the AE and FillMask models in emphasizing the benefit of wider context in LLM-based communication UIs. However, note that SpeakFaster provided higher KSRs than Gboard's forward word prediction even when no wider conversational context was available.

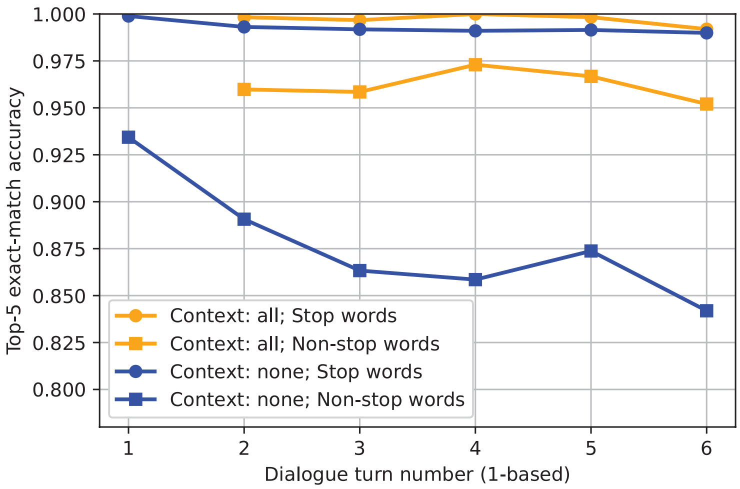

Supplementary Figure 2. Exact-match accuracy of the fine-tuned LaMDA for FillMask task, evaluated on the test split of the TDC corpus, under different amounts of dialogue context for predicting masked stop words and non-stop words. The results are evaluated on the same sentences used for the evaluation of the fine-tuned LaMDA for abbreviation expansion. The results are plotted similarly to Supplementary Figure 1 Panel B. In each set of blue or orange curves, a curve is shown for the accuracy of predicting masked stop words and non-stop words, respectively.

3 Serving of large language models

During the simulations and user studies, for both KeywordAE and FillMask, the fine-tuned LLMs were served with 16 Google TPU v3 chips[8] in a 4x4 configuration. To increase inference speed, we performed post-training quantization to the bfloat16 dtype on both the KeywordAE and FillMask LaMDA models. The served KeywordAE and FillMask models were configured to a maximum input length of 128 SentencePiece tokens[7], which was sufficient to accommodate up to five contextual dialogue turns, each consisting of ten or fewer words for all the test dialogues used in the lab study with LP1. SentencePiece tokens are variable length tokens learned from the training corpus. Short and frequent words such as "the" and "time" are single tokens, while longer and less frequent tokens such as "understanding" are split as two or more tokens. The AE model was served with a maximum decoder step of 20 tokens, which is sufficient to capture the vast majority of phrases with ten or fewer words and mid-sentence punctuation. To serve the FillMask model, however, we exploited the fact that the output of the model is a single word and hence could use a smaller value for maximum decoder steps (6) to achieve a shorter serving latency.

4 User study design and details

No analysis on the gender basis was carried out on the user study data as the text-entry behavior is not expected to vary in systematic ways between genders.

The non-AAC and eye-gaze user studies were based on the same user-interface code base, available in open source at https://github.com/TeamGleason/SpeakFaster/tree/main/webui.

The ten dialogues used in the scripted blocks of the lab study were selected from the test split of the Turk Dialogues dataset11, hence were not seen by the LLMs during their fine-tuning for KeywordAE or FillMask. We selected the dialogues based on the criteria of: 1) consisting of exactly one sentence per dialogue turn, 2) each sentence contains ten or fewer words, and 3) contain no potentially offensive or inappropriate content. The IDs of the ten dialogues selected dialogues were: chain2_633_298_601_4_948_1016, chain2_431_1021_754_865_533_290, chain3_242_108_214_13_28_50, chain2_450_34_1143_868_23_220, chain2_2121_755_43_246_381_709, chain2_752_401_1278_363_1136_1091, chain2_1578_1057_1229_143_692_855, chain2_58_150_868_489_383_264, chain2_58_150_868_489_383_264, and chain3_112_258_148_288_146_242, which can be cross reference with the raw dataset at https://aactext.org/turk/turk/turk-dialogues.txt. In the dialogue with ID chain3_242_108_214_13_28_50, we fixed a grammatical error in the 5th turn, "I've tried too" → "I've tried to". Eight additional dialogues that satisfy these criteria were selected for practice purposes. Their IDs were chain2_230_210_1152_921_190_391, chain2_1854_397_1176_1052_141_408, chain3_161_342_288_171_313_222, chain2_888_773_852_406_924_605, chain3_45_147_301_192_331_261, chain2_1481_1455_10_53_610_18, chain2_1052_582_996_1165_267_929, chain2_839_1478_209_43_1020_495.

The unscripted dialogues were prompted with the following starter questions:

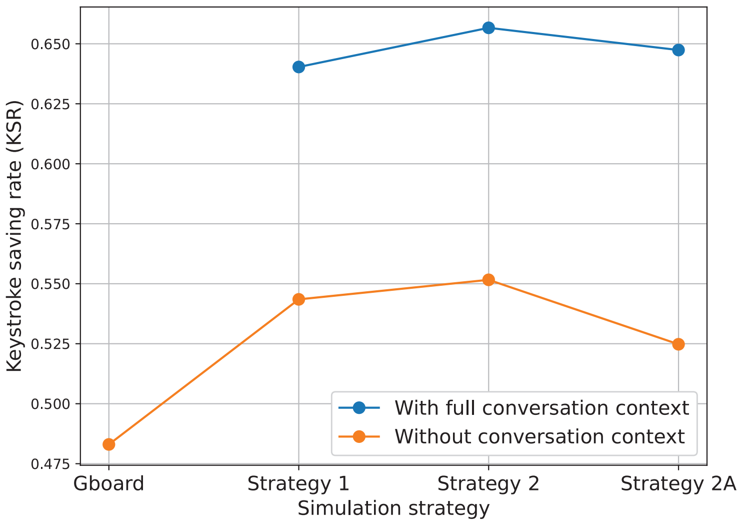

Supplementary Figure 3. Comparing the keystroke saving rate (KSR) from simulations ran on three different use strategies of SpeakFaster as well as the Gboard baseline. The results shown in this figure are based on presenting top-5 options from AE, FillMask, and Gboard word suggestions. The blue curve shows the KSR values obtained from full utilization of conversation context by the AE and FillMask models of SpeakFaster, i.e., all the previous turns of a dialogue. It does not contain data for Gboard because Gboard's n-gram model does not utilize the previous dialogue turns. The orange curve shows the results obtained from not utilizing the conversation context.

- "How do you like the weather today?"
- "What did you do yesterday?"
- "What pets have you had?"
- "What kind of books do you read?"
- "What kind of music do you listen to?"
- "What sports do you like to watch?"
- "Where would you like to go for vacation?"
- "Do you like the city you live in?"

The lab study participant was instructed to respond to these questions with dialogue turns that each consisted of a single sentence with ten or fewer words. Each unscripted dialogues lasted six turns (i.e., the same as the scripted ones), in which the participant was the interlocutor in the 2nd, 4th, and 6th turns, while the experimenter performed the 3rd and 5th turns impromptu.

Supplementary Figure 4 shows the protocol of the lab study with the the non-AAC participants and the eye-gaze participant LP1. It was arranged in three blocks, with interspersed breaks. Each block contained five six-turn dialogues, the first and last of which were the baseline condition of using the on-screen soft keyboard Gboard (for the non-AAC participants) or the Tobii eye-gaze keyboard (for LP1), while the three dialogues in the middle were based on the SpeakFaster UI. When the participants were tested on the SpeakFaster UI, they still used Gboard or the Tobii eye-gaze keyboard to enter the characters for the initial abbreviations and the characters subsequently added to the abbreviations to form incomplete or complete keywords. The first and second blocks used scripted dialogues from the test split of the TDC corpus. They differed in whether the participant played the role of the first or second interlocutor. The third block was based on unscripted dialogues in which the participant responded in the 2nd turn to the starter questions listed above, in addition to following up with the experiment in the 4th and 6th turns.

Supplementary Figure 5 shows how the lab study user LP1 gradually improved in the efficiency of text entry in the SpeakFaster UI through initial practice prior to the actual lab study over two days, in comparison with the same learning curve from averaging the 19 non-AAC lab-study participants. It can be seen that despite showing a slower learning rate, approximately ten practice dialogues were sufficient to train LP1 beyond the initial slow rate (< 5 WPM) and reach a level > 10

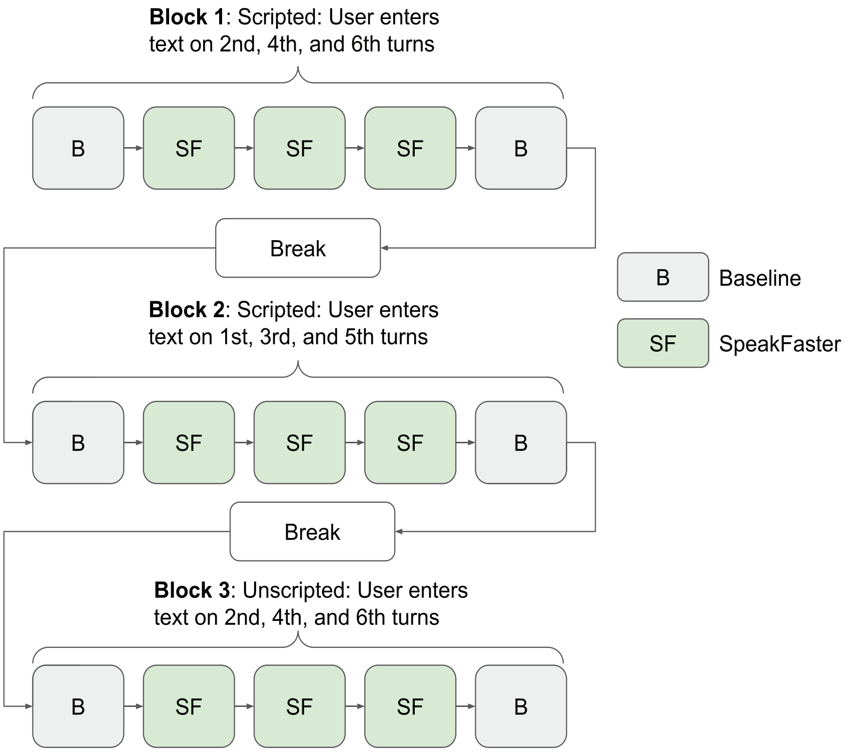

Supplementary Figure 4. Design of the lab study with participant LP1. Each box shows a six-turn dialogue used for testing. The gray and green boxes shows trials of the baseline condition and the SpeakFaster condition, respectively.

WPM. This shows that for eye-gaze communication users with cognitive abilities within normal limits at least, SpeakFaster has a manageable learning curve.

The following dialogue turns from the non-AAC user study participants were excluded: six dialogue turns (from three participants) due to temporary LLM server outage that occurred during the study sessions, a single turn due to the participant's mistake in entering partner's dialogue turn as their own turn, and another single due to an unsupported abbreviated word that mixed letters with numbers. For the AAC lab and field studies, only a single dialogue turn from the participant LP1 was excluded due to temporary LLM server outage.

Supplementary Figure 5 shows how the lab study user LP1 gradually improved in the efficiency of text entry in the SpeakFaster UI through initial practice prior to the actual lab study over two days, in comparison with the same learning curve from averaging the 19 non-AAC lab-study participants. It can be seen that despite showing a slower learning rate, approximately ten practice dialogues were sufficient to train LP1 beyond the initial slow rate (< 5 WPM) and reach a level > 10 WPM. This shows that for eye-gaze communication users with cognitive abilities within normal limits at least, SpeakFaster has a manageable learning curve.

The following dialogue turns from the non-AAC user study participants were excluded: six dialogue turns (from three participants) due to temporary LLM server outage that occurred during the study sessions, a single turn due to the participant's mistake in entering partner's dialogue turn as their own turn, and another single due to an unsupported abbreviated word that mixed letters with numbers. For the AAC lab and field studies, only a single dialogue turn from the participant LP1 was excluded due to temporary LLM server outage.

5 Accuracy and speed trade-off

Adopting SpeakFaster's abbreviation expansion resulted in significant speed ups, but in some cases it reduced accuracy. For the lab study user LP1, a third (six) of the 18 scripted dialogue turns performed with the SpeakFaster UI contained errors, i.e., mismatches between the entered sentence and the sentence prompt. This led to an average word error rate (WER) of 15.4% across LP1's SpeakFaster turns, which was higher than the word error rate observed on the same participant under the 12 dialogue turns of baseline (non-SpeakFaster) condition: 0%. However, as determined by the speech-language pathologists on our research team, only two of the errors were categorized "major", i.e., affects the meaning of comprehensibility of the text. The remaining four errors were categorized as "minor" errors that did not affect the meaning of the sentence or its

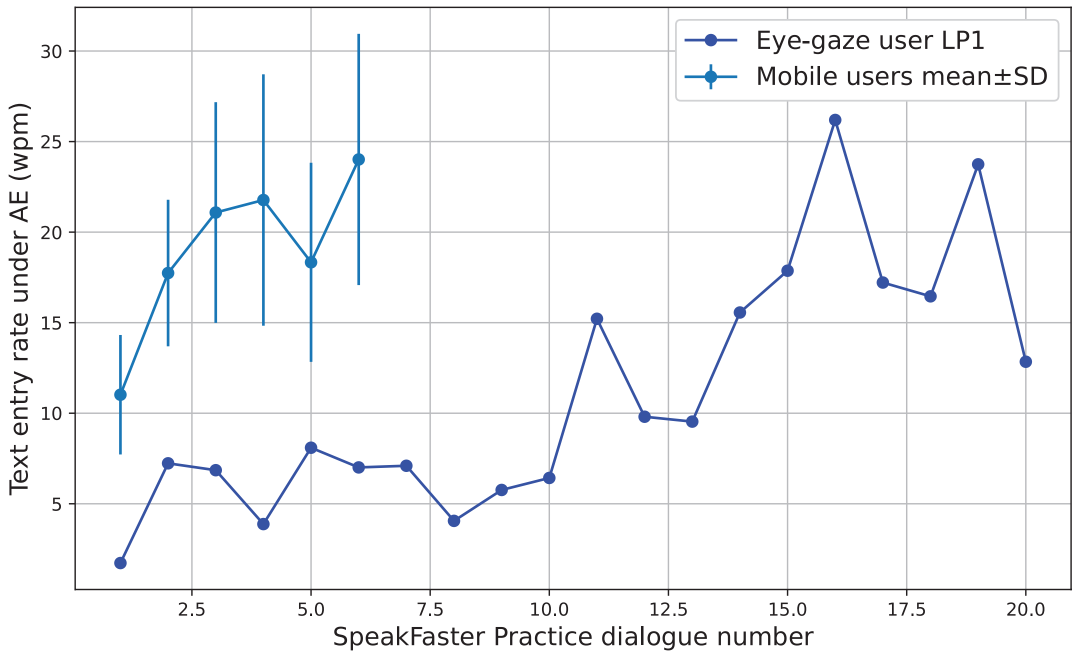

Supplementary Figure 5. Learning curve of eye-gaze user LP1 on the SpeakFaster text-entry paradigm over 20 practice dialogues, in comparison with the average learning curve of the 19 users in the non-AAC touch-typing study. The error bars in the curve for mobile users are standard deviations (SDs) over all N=19 participants. In LP1, the practice was conducted over two separate days before the lab study.

comprehension in a significant way, but may have resulted in incorrect grammar or idiom. This indicates that a majority of the word errors seen in the SpeakFaster-entered phrases did not hinder the communication in terms of intentions and meaning, although it may still impact the perception of the user's communication or cognitive abilities in undesirable ways. Future studies can explore automatic correction[9] of such errors by leveraging LLMs and context awareness.

6 Temporal, Cost, and Error Analyses of Text Entry

When analyzing the results from offline simulations and event logs from user studies, we used the definition of keystroke saving rate (KSR):

$$KSR = 1 - \frac{N_a}{N_c} \tag{1}$$

where N_c is the character length of the entered phrase and N_a is the number of total keystrokes and UI actions that the user performs to enter the phrase. The keystrokes include the ones used to enter the abbreviation, the ones used to specify keywords (complete or incomplete), and the ones used to type the words that cannot be found with FillMask. The UI interactions include the clicks used to enter the Spelling or FillMask mode, the clicks used to specify which word to spell, the clicks that specify which word to get replacements for through FillMask, in addition to the clicks used to select matching results from KeywordAE and FillMask. The actions that select the final phrase for text-to-speech output (e.g., gaze-clicking the speaker buttons in Fig. 1) are not included in the KSR calculation.

7 Data analysis tools

We used a combination of open source and non-open source software for data analysis. The open source libraries we used include: numpy (1.26.0), scipy (1.9.3), pandas (1.5.3), matplotlib (3.6.1), and statsmodel (0.12.2).

References

1. Caligari, M., Godi, M., Guglielmetti, S., Franchignoni, F. & Nardone, A. Eye tracking communication devices in amyotrophic lateral sclerosis: impact on disability and quality of life. *Amyotrophic Lateral Sclerosis and Frontotemporal Degeneration* **14**, 546–552 (2013).

2. Kane, S. K., Morris, M. R., Paradiso, A. & Campbell, J. " at times avuncular and cantankerous, with the reflexes of a mongoose" understanding self-expression through augmentative and alternative communication devices. In *Proceedings of the 2017 acm conference on computer supported cooperative work and social computing*, 1166–1179 (2017).

3. Vertanen, K. Towards improving predictive aac using crowdsourced dialogues and partner context. In *Proceedings of the 19th International ACM SIGACCESS Conference on Computers and Accessibility*, 347–348 (2017).

4. Vertanen, K. & Kristensson, P. O. The imagination of crowds: conversational aac language modeling using crowdsourcing and large data sources. In *Proceedings of the 2011 Conference on Empirical Methods in Natural Language Processing*, 700–711 (2011).

5. Li, Y. *et al.* Dailydialog: A manually labelled multi-turn dialogue dataset. *arXiv preprint arXiv:1710.03957* (2017).

6. Danescu-Niculescu-Mizil, C. & Lee, L. Chameleons in imagined conversations: A new approach to understanding coordination of linguistic style in dialogs. *arXiv preprint arXiv:1106.3077* (2011).

7. Kudo, T. & Richardson, J. Sentencepiece: A simple and language independent subword tokenizer and detokenizer for neural text processing. *arXiv preprint arXiv:1808.06226* (2018).

8. Jouppi, N. P. *et al.* In-datacenter performance analysis of a tensor processing unit. In *Proceedings of the 44th annual international symposium on computer architecture*, 1–12 (2017).

9. Bryant, C. *et al.* Grammatical error correction: A survey of the state of the art. *Computational Linguistics* 1–59 (2023).
