# Supplementary material for: Using large language models to accelerate communication for eye gaze typing users with ALS
Source: Nat Commun. 2024 Nov 1;15:9449. doi: 10.1038/s41467-024-53873-3 (PMC11530652; doi:10.1038/s41467-024-53873-3)
Supplement: Supplementary file 2 — Reporting Summary [file 41467_2024_53873_MOESM2_ESM.pdf]

Corresponding author(s): Shanqing Cai

Last updated by author(s): Aug 9, 2024

Reporting Summary

Nature Portfolio wishes to improve the reproducibility of the work that we publish. This form provides structure for consistency and transparency in reporting. For further information on Nature Portfolio policies, see our Editorial Policies and the Editorial Policy Checklist.

Statistics

For all statistical analyses, confirm that the following items are present in the figure legend, table legend, main text, or Methods section.

n/a	Confirmed	
☐	☒	The exact sample size (*n*) for each experimental group/condition, given as a discrete number and unit of measurement
☐	☒	A statement on whether measurements were taken from distinct samples or whether the same sample was measured repeatedly
☐	☒	The statistical test(s) used AND whether they are one- or two-sided<br>*Only common tests should be described solely by name; describe more complex techniques in the Methods section.*
☐	☒	A description of all covariates tested
☐	☒	A description of any assumptions or corrections, such as tests of normality and adjustment for multiple comparisons
☐	☒	A full description of the statistical parameters including central tendency (e.g. means) or other basic estimates (e.g. regression coefficient) AND variation (e.g. standard deviation) or associated estimates of uncertainty (e.g. confidence intervals)
☐	☒	For null hypothesis testing, the test statistic (e.g. *F*, *t*, *r*) with confidence intervals, effect sizes, degrees of freedom and *P* value noted<br>*Give P values as exact values whenever suitable.*
☒	☐	For Bayesian analysis, information on the choice of priors and Markov chain Monte Carlo settings
☒	☐	For hierarchical and complex designs, identification of the appropriate level for tests and full reporting of outcomes
☐	☒	Estimates of effect sizes (e.g. Cohen's *d*, Pearson's *r*), indicating how they were calculated

Our web collection on statistics for biologists contains articles on many of the points above.

Software and code

Policy information about availability of computer code

Data collection	We used custom software for the user interface (UI) of SpeakFaster, which is available in the open source repository on GitHub (https://github.org/TeamGleason/SpeakFaster). The large language models (LLMs) backend is based on Google's Proprietary LaMDA model. However, the code for training, evaluation, and serving of the LLMs is not open source.
Data analysis	We used a combination of open source and non-open source software for data analysis. The open source libraries we used include: numpy (1.26.0), scipy (1.9.3), pandas (1.5.3), matplotlib (3.6.1), statsmodels (0.12.2),

For manuscripts utilizing custom algorithms or software that are central to the research but not yet described in published literature, software must be made available to editors and reviewers. We strongly encourage code deposition in a community repository (e.g. GitHub). See the Nature Portfolio guidelines for submitting code & software for further information.

Data

Policy information about availability of data

All manuscripts must include a data availability statement. This statement should provide the following information, where applicable:

- Accession codes, unique identifiers, or web links for publicly available datasets
- A description of any restrictions on data availability
- For clinical datasets or third party data, please ensure that the statement adheres to our policy

Data used to train and evaluate the LLMs in this paper are available from existing public sources. We list the training datasources and code for processing them in

the data directory of the TeamGleason SpeakFaster GitHub repository:https://github.com/TeamGleason/SpeakFaster/tree/main/data/naacl_2022_suppl_data. The stimuli usedin the user study are available in Supplementary Information. The raw data from the user studies are protected and arenot available due to data privacy laws. The processed data based on the raw user study data that are presented in thefigures and table of this article are available for download at the same GitHub repository as mentioned above:https://github.com/TeamGleason/SpeakFaster/tree/main/data/user_study_paper_data.

Research involving human participants, their data, or biological material

Policy information about studies with human participants or human data. See also policy information about sex, gender (identity/presentation), and sexual orientation and race, ethnicity and racism.

Reporting on sex and gender	The gender distribution of all 19 non-AAC user study participants and two AAC user study participants are reported in the manuscript. The 19 non-AAC user study participants are approximately balanced in gender between male (9) and female (10).
Reporting on race, ethnicity, or other socially relevant groupings	Although we do not report the race and ethnicity of the participants, the relevant linguistic characteristics of the participants are reported in the manuscript. This is specifically the American English speaking attribute of the 19 non-AAC user study participants, the native American English speaking attribute of the two AAC user study participants. The manuscript also contains information about the level of education of all these participants.
Population characteristics	The covariate-relevant characteristics, including level of education, language attributes, and the experience in using eye-gaze AAC (for the AAC user study participants) are reported in the manuscript and mentioned elsewhere in this Reporting Summary.
Recruitment	As stated above, the non-AAC user study participants were recruited via email advertisement. This is not expected to introduce a strong self-selection bias. The AAC user study participants were experienced users of eye-gaze text entry with cognitive abilities within normal bounds who use the technology on a daily basis. As we pointed out in the Discussion section of the article, this may have introduced a bias of the results towards experienced eye-gaze AAC users and calls for future follow-up studies with a larger and more diverse participant cohort.
Ethics oversight	This study was conducted consistent with user research studies for Google's consumer products (https://userresearch.google.com/) and accessibility trusted tester program (e.g.https://docs.google.com/forms/d/e/1FAIpQLSfcb-l0mCZ__09SSyFAuI_k2WBLR05URYbR_Stv9N42u7GTiw/viewform). Additionally, we sought and received approval by the ethical review committees of Google and Team Gleason.

Note that full information on the approval of the study protocol must also be provided in the manuscript.

Field-specific reporting

Please select the one below that is the best fit for your research. If you are not sure, read the appropriate sections before making your selection.

☐ Life sciences ☒ Behavioural & social sciences ☐ Ecological, evolutionary & environmental sciences

For a reference copy of the document with all sections, see nature.com/documents/nr-reporting-summary-flat.pdf

Behavioural & social sciences study design

All studies must disclose on these points even when the disclosure is negative.

Study description	User studies testing the usability and user performance in a novel software user interface for text entry, for both the non-AAC use case on a mobile device and AAC user case based on eye tracking. The design of the study is primarily quantitative, involving dependent variables including text-entry rate (words per minute), keystroke saving rate, various timing metrics such as inter-key intervals, and their correlation with offline simulation results. Details are provided in the manuscript.
Research sample	As detailed in the manuscript, the 19 non-AAC study participants were a mixture of employees of Google's Cambridge, Massachusetts office and volunteers recruited from the Boston area. The two AAC study participants were an opportunistic cohort.
Sampling strategy	The 19 non-AAC user study participants were recruited via email advertisement. The size of this group was determined as sufficient based on the typical group sizes reported in previous published user studies of text entry (e.g., Xu et al. 2019, Li et al. 2023).<br><br>As stated above, the two AAC study participants were recruited through convenience sampling. Given the recruitment of a large number of AAC users as study participants was difficult, the statistical power was determined as sufficient at a within-subject level by ensuring sufficient number of trials for each AAC participant.<br><br>References:<br>* Xu, Z., Wong, P. C., Gong, J., Wu, T. Y., Nittala, A. S., Bi, X., ... & Yang, X. D. (2019, October). Tiptext: Eyes-free text entry on a fingertip keyboard. In Proceedings of the 32nd Annual ACM Symposium on User Interface Software and Technology (pp. 883-899).<br>* Li, T., Quinn, P., & Zhai, S. (2023). C-PAK: correcting and completing variable-length prefix-based abbreviated keystrokes. ACM Transactions on Computer-Human Interaction, 30(1), 1-35.
Data collection	Detailed data collection methodology, including the software, user interface, pre-data collection introduction and practice, along with detailed study design and protocol are included in the manuscript.

	For each of the 19 non-AAC user study sessions, only one or two experimenters (S.C. and K.S. in the author list) were present with the participant. For the AAC lab study sessions, only one or two experimenters (S.C and K.S. in the author list) were present with the participant. For the AAC field study, as the settings was real life, the participant was present with various conversation partners across the data collection period from November 2021 to May 2022, including the experimenters on some days.<br><br>The experimenters were not blinded to the study condition. The rationale was that for the scripted dialogues, the awareness on the part of the experimenter could not have affected the participants' text entry behavior in significant ways. For the unscripted dialogues, the randomization of the predefined starter questions alleviations the potential concern of experimenter conversation content affects the participants' text entry behavior significantly.
Timing	All study sessions were conducted in the time period between 2021 and late 2022. Specifically, the 19 non-AAC user study sessions were conducted between 2022-09-13 and 2022-10-28. The AAC lab study participant was tested on two days: 2022-10-27 and 2022-10-28. The data from the AAC field study participant was collected between in a period between November 2021 and May 2022.
Data exclusions	The manuscript contains a paragraph describing the small number of (six) dialogue turns excluded from participants due to temporary LLM server outage and user mistakes unrelated to the text entry method that occurred during the user study sessions.
Non-participation	There were no dropped participants in either the non-AAC or AAC user studies.
Randomization	The non-AAC users were randomly assigned to the two posture groups. The order of the scripted dialogues were randomized among all 21 study participants. The order of the the two types of dialogues (user starting with the first turn and the user starting with the second turn) were randomized and counterbalanced among all the non-AAC and AAC study participants.

Reporting for specific materials, systems and methods

We require information from authors about some types of materials, experimental systems and methods used in many studies. Here, indicate whether each material, system or method listed is relevant to your study. If you are not sure if a list item applies to your research, read the appropriate section before selecting a response.

Materials & experimental systems

n/a	Involved in the study
☒	☐ Antibodies
☒	☐ Eukaryotic cell lines
☒	☐ Palaeontology and archaeology
☒	☐ Animals and other organisms
☒	☐ Clinical data
☒	☐ Dual use research of concern
☒	☐ Plants

Methods

n/a	Involved in the study
☒	☐ ChIP-seq
☒	☐ Flow cytometry
☒	☐ MRI-based neuroimaging

Plants

Seed stocks	N/A
Novel plant genotypes	N/A
Authentication	N/A
